# Correction to “Epidemiological characteristics and risk factors for heart failure in Japanese patients with type 2 diabetes: A retrospective analysis of the J‐DREAMS database”

**DOI:** 10.1111/jdi.70422

**Published:** 2026-08-18

Ohsugi M , Nitta D , Naito Y , Ueki K , Epidemiological characteristics and risk factors for heart failure in Japanese patients with type 2 diabetes: A retrospective analysis of the J‐DREAMS database. J Diabetes Investig 2025; 16: 414–425. 10.1111/jdi.14378.PMC1187138639853963

The entry “Approval of the research protocol: N/A” is an error. The approval of the research protocol is provided below.

Approval of the research protocol: The study protocol was reviewed and approved by the Institutional Review Board of the National Center for Global Health and Medicine (approval number: NCGM‐S‐004440‐00).

We apologize for this error.

